# Immunomodulatory activities of pixatimod: emerging nonclinical and clinical data, and its potential utility in combination with PD-1 inhibitors

**DOI:** 10.1186/s40425-018-0363-5

**Published:** 2018-06-14

**Authors:** Edward Hammond, Nicole M. Haynes, Carleen Cullinane, Todd V. Brennan, Darryn Bampton, Paul Handley, Tomislav Karoli, Fleur Lanksheer, Liwen Lin, Yiping Yang, Keith Dredge

**Affiliations:** 1 Zucero Therapeutics, Brisbane, QLD 4076 Australia; 2 Division of Cancer Research, Peter MacCallum Cancer Centre, Melbourne, VIC 3000 Australia; 3 Sir Peter MacCallum Department of Oncology, University of Melbourne, Parkville, VIC 3052 Australia; 4 Department of Surgery, Duke University Medical Center, Durham, North Carolina 27710 USA; 5 Progen Pharmaceuticals, Brisbane, QLD 4076 Australia; 6 Departments of Medicine and Immunology, Duke University Medical Center, Durham, North Carolina 27710 USA; 7 Present address: Novasep, Kalkstrasse 218, 51377 Leverkusen, Germany; 8 Present address: School of Humanities and Social Science, The University of Newcastle, Newcastle, NSW Australia

**Keywords:** Pixatimod, PG545, Immunomodulatory, Tumor-associated macrophage, Dendritic cell, NK cell, T cell, PD-1 inhibition, Toxicology, Pharmacokinetics, Clinical trial, Pancreatic adenocarcinoma

## Abstract

**Background:**

Pixatimod (PG545) is a novel clinical-stage immunomodulatory agent capable of inhibiting the infiltration of tumor-associated macrophages (TAMs) yet also stimulate dendritic cells (DCs), leading to activation of natural killer (NK) cells. Preclinically, pixatimod inhibits heparanase (HPSE) which may be associated with its inhibitory effect on TAMs whereas its immunostimulatory activity on DCs is through the MyD88-dependent TLR9 pathway. Pixatimod recently completed a Phase Ia monotherapy trial in advanced cancer patients.

**Methods:**

To characterize the safety of pixatimod administered by intravenous (IV) infusion, a one month toxicology study was conducted to support a Phase Ia monotherapy clinical trial. The relative exposure (AUC) of pixatimod across relevant species was determined and the influence of route of administration on the immunomodulatory activity was also evaluated. Finally, the potential utility of pixatimod in combination with PD-1 inhibition was also investigated using the syngeneic 4T1.2 breast cancer model.

**Results:**

The nonclinical safety profile revealed that the main toxicities associated with pixatimod are elevated cholesterol, triglycerides, APTT, decreased platelets and other changes symptomatic of modulating the immune system such as pyrexia, changes in WBC subsets, inflammatory changes in liver, spleen and kidney. Though adverse events such as fever, elevated cholesterol and triglycerides were reported in the Phase Ia trial, none were considered dose limiting toxicities and the compound was well tolerated up to 100 mg via IV infusion. Exposure (AUC) up to 100 mg was considered proportional with some accumulation upon repeated dosing, a phenomenon also noted in the toxicology study. The immunomodulatory activity of pixatimod was independent of the route of administration and it enhanced the effectiveness of PD-1 inhibition in a poorly immunogenic tumor model.

**Conclusions:**

Pixatimod modulates innate immune cells but also enhances T cell infiltration in combination with anti-PD-1 therapy. The safety and PK profile of the compound supports its ongoing development in a Phase Ib study for advanced cancer/pancreatic adenocarcinoma with the checkpoint inhibitor nivolumab (Opdivo®).

**Trial registration:**

ClinicalTrials.gov Identifier: NCT02042781. First posted: 23 January, 2014 - Retrospectively registered.

**Electronic supplementary material:**

The online version of this article (10.1186/s40425-018-0363-5) contains supplementary material, which is available to authorized users.

## Background

**Fig. 1:**
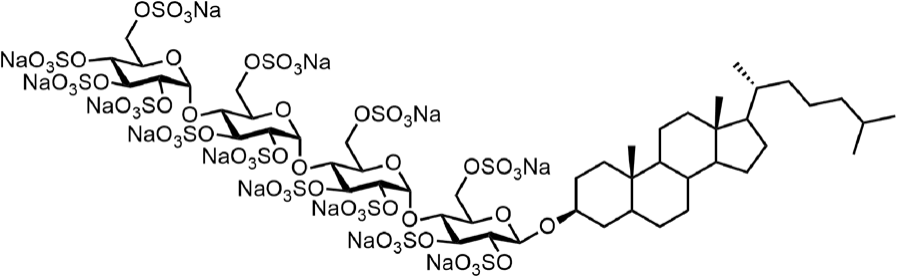
The structure of pixatimod, formerly known as PG545

Pixatimod is the international non-proprietary name designated to the compound formerly described as PG545 in the literature [1] and is a cholestanol-sulfotetrasaccharide conjugated small molecule compound (Fig. 1). The oligosaccharide backbone of pixatimod is derived from starch, and retains the amylose structure of α(1 → 4)-linked glucose residues. Coupling the sulfated oligosaccharide to a lipophilic cholestanol aglycone significantly increased the elimination half-life in vivo, while reducing the unwanted anticoagulant activity associated with similar compounds [2] but retaining the potent inhibition of the heparan sulfate (HS)-degrading enzyme heparanase-1 (HPSE), a key drug target [1, 3, 4] considered a master regulator of the aggressive cancer phenotype [5–8].

Pixatimod inhibits the infiltration of tumor-associated macrophages (TAMs) [9, 10] but, moreover, it also stimulates dendritic cells (DCs) [11]. In terms of its immunomodulatory activity on TAMs, there is preclinical evidence that heparanase may be responsible for this activity [10] and is known to direct the tumor-promoting behaviour of TAMs in pancreatic cancer [12], and promote disease progression in pancreatitis [13, 14] and pancreatic cancer [14–16]. The presence of TAMs and M2 macrophages limit immune cell engagement and are associated with decreased survival in pancreatic cancer [17]. However, M1, but not M0 or M2 macrophages, have the ability, not unlike DCs, to prime autologous NK cells and direct T cells [18, 19]. In addition to the reported activity of pixatimod on TAMs and M2 macrophages [9, 10], the compound also exerts a strong immunostimulatory activity on CD11c^+^ DCs, via toll-like receptor 9 (TLR9) and IL-12 leading to activation of IFN-γ producing natural killer (NK) cells [11]. As M1 macrophages also express CD11c, TLR9 and produce IL-12 [20], it is plausible that these myeloid cells play a central role in the activation of innate immunity by pixatimod. Clearly, pixatimod’s immunomodulatory effects on these myeloid cells enhance innate immunity and may also drive adaptive immune responses depending on the context (e.g. presence of tumor antigens, combination with PD-1 inhibitors).

Pixatimod has been shown to potently inhibit solid tumor progression and metastasis in a number of syngeneic, orthotopic and xenograft murine models of cancer either alone [1, 10, 21–28] or in combination with chemotherapy such as paclitaxel or gemcitabine [29, 30] and cyclophosphamide with the latter being considered to be mediated by NK cell activation [11]. But its utility in combination with immune checkpoint blockade and the potential to enhance T cell function or number of infiltrating T cells into the tumor microenvironment (TME) has not been reported.

Initial clinical development of pixatimod used the subcutaneous route (SC) but switched to intravenous (IV) infusion following local injection site reactions [29]. Subsequently, a non-rodent toxicology study (in beagle dogs) was part of the nonclinical data generated to support the new route of administration which was successfully utilized in a recently completed Phase Ia monotherapy study [31]. An investigational new drug (IND) application was successfully lodged with the USFDA in 2016.

Herein, we describe the new research and development of pixatimod as a once-weekly IV infusion for the treatment of cancer, revealing new data on the proposed mechanism of action, the potential utility of pixatimod in combination with a PD-1 inhibitor, the toxicology and comparative pharmacokinetic profile of pixatimod, and discuss the current clinical and regulatory status of this unique immunomodulatory agent.

## Methods

### Nonclinical and clinical safety

A 1 month toxicology study of pixatimod in beagle dogs was performed under OECD Good Laboratory Practice (GLP) to assess the toxicity and toxicokinetic profile of pixatimod with 5 intravenous, short term (approximately 1 h) infusions at three defined dose levels of 2.5, 7.5 and 20 mg/kg weekly over 29 days. Blood samples were collected for toxicokinetic investigation to provide information on the systemic exposure. Parameters monitored included mortality and morbidity, clinical signs, food consumption, body weight and body temperature, ophthalmoscopy and electrocardiographic measurements. Laboratory investigations were performed using haematology (ADVIA 120), coagulation (AMAX Density Plus Coagulometer), clinical chemistry (VITROS 950) and urinalysis (URYXXON 300). At the end of the treatment, all animals were euthanized and subjected to a complete necropsy with selected organs weighed and followed by a detailed histopathology evaluation. Bone marrow smears from the femur and sternum were prepared at necropsy, fixed and stained with May-Grünwald and Giemsa stain for analysis. Blood samples obtained during the Phase Ia monotherapy study (PG545102) were collected weekly and the parameters reported herein were analysed by local hospital laboratories.

### Nonclinical and clinical bioanalysis and pharmacokinetics

Plasma samples were analysed using a LC-MS/MS method as previously described [21]. Pharmacokinetic parameters in animal studies were determined by non-compartmental analyses (NCA) of the mean plasma pixatimod concentration vs time profiles for each dose using the linear trapezoidal method in WinNonlin 5.2.1. For comparison with previous animal studies, exposure data (AUC_0-last_) in clinical samples were derived using individual subject NCA type exposure parameters from the original plasma concentration data using R (64-bit) Version 3.0.1.

### Nonclinical efficacy studies

Female Balb/c mice (6–8 weeks) were obtained from the Walter & Eliza Hall Institute (Melbourne, Australia). Animal experiments were performed in accordance with institutional guidelines of the Peter MacCallum Cancer Centre. To determine the utility of pixatimod in combination with a PD-1 checkpoint inhibitor antibody (clone RMP1–14 or isotype control antibody 2A3, Bio-X-Cell, NH, USA), mice were inoculated with 1 × 10^5^ 4T1.2 cells into the mammary fatpad. One week later, mice with similar sized tumors (mean tumor volume 56 mm^3^) were randomised into four groups (*n* = 6 mice per group): saline + isotype antibody, pixatimod + isotype antibody, saline + anti-PD-1 antibody and pixatimod + anti-PD-1 antibody. Pixatimod was administered at 15 mg/kg IP weekly for 3 weeks (days 1, 8 and 15) and anti-PD-1 or isotype antibody (200 μg) were given IP on days 1, 4, 8, 11 and 15. The experiment was ended on day 18 post treatment initiation (25 days post inoculation) due to emerging toxicities in all treatment groups (e.g. piloerection). Satellite groups of mice (*n* = 4 mice per group) received the same treatments but were euthanized on day 11 for the ex vivo analysis of the immune microenvironment of the 4T1.2 tumors. Spleens were also removed and used as background staining controls.

Immune cells from the collagenase (Collagenase IV, Worthington Biochemical Corporation, NJ, USA) processed tumors and spleens were analysed by flow cytometry using an LSR II analyser (BD Biosciences). Antibodies (ThermoFisher Scientific) used to assess the T cells and NK cell compartments of the treated 4T1.2 tumors and spleens include CD45.2 (clone 104), TCRb (clone H57–597), CD4 (clone GK1.5), CD8 (clone 53–6.7), CD44 (clone IM7), CD62L (clone MEL-14), CD69 (clone H1.2F3), CD49b (clone DX5), CD27 (clone LG.7F9), CD335 (clone 29A1.4), DAPI.

To investigate the effect that pixatimod route of administration has upon the activity of this compound, C57BL/6 mice were treated with pixatimod 20 mg/kg intraperitoneally (IP), intravenously (IV) or subcutaneously (SC) and 2 days later, spleens were isolated to study the activation levels of NK cells for surface expression of CD69 or intracellular expression of IFN-γ. Antibodies were CD3ε (145-2C11), NK1.1 (PK136), CD69 (H1.2F3), IFN-γ (XMG1.2), hamster IgG1 isotype (G235–2356) and rat IgG1 isotype (R3–34) were from BD Biosciences (San Jose, CA, USA). Intracellular staining for IFN-γ was performed following ex vivo stimulation of splenocytes for 4 h with 20 ng/ml PMA and 50 ng/ml ionomycin in the presence of 5 μg/mL brefeldin A. Intracellular staining for IFN-γ was performed following treatment with Cytofix/Cytoperm (BD Biosciences) solution. Flow cytometric data were acquired using a FACSCanto flow cytometer (BD Biosciences), and events were analysed using FlowJo Version 9.9.6 software (TreeStar, Ashland, OR, USA).

### Statistical analysis

In the 4T1.2 model, the percentage tumor growth inhibition was determined according to the following formula: 100 × (1-ΔT/ ΔC) where ΔC and ΔT were calculated by subtracting the mean tumor volume in each group on day 1 of treatment from the mean tumor volume on the day of analysis. Statistical analysis was performed using GraphPad Prism, v 6.0 (GraphPad, La Jolla, CA). An ANOVA analysis was performed followed by Dunnett’s post hoc test to compare the tumor growth in the treated groups to the vehicle control. In the dog toxicology study, the analysis was performed using non-parametric Kruskal-Wallis test. The frequency of clinical observations, and necropsy and histopathology findings, was calculated as applicable. For all statistical analyses, statistically significance differences between control and treatment groups was signified by *P < 0.05, **P < 0.01, ***P < 0.001, ****P < 0.0001 versus vehicle control.

## Results

### Nonclinical and clinical safety

**Fig. 2:**
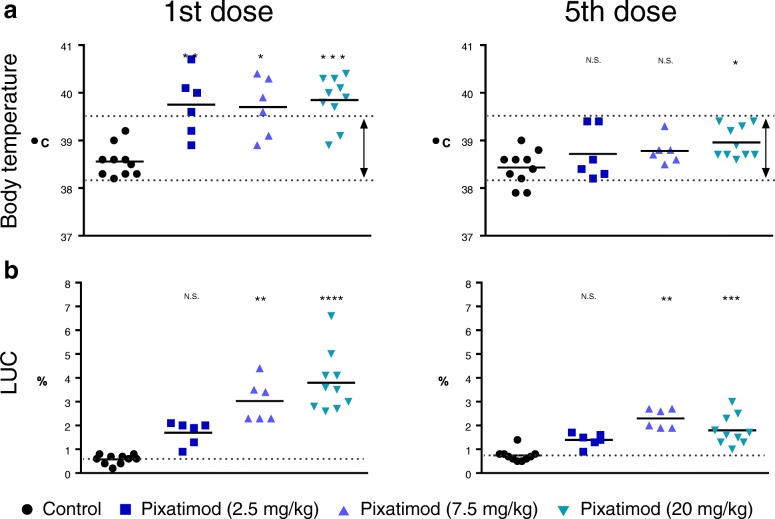
Effect on body temperature and large unstained cells (LUC) following a single IV infusion of pixatimod in beagle dogs. **a** Body temperature of treated dogs measured 1 day after the first dose and after the 5th dose of pixatimod (weekly dosing). **b** LUC measured in blood 2 days after the first dose and after the 5th dose of pixatimod. Treatment averages indicated with short solid horizontal lines. Dotted lines represent either the mean value of the control group (LUC) or the normal temperature range for beagle dogs. *P < 0.05, **P < 0.01, ***P < 0.001, ****P < 0.0001 versus control (Kruskal-Wallis test)

**Fig. 3:**
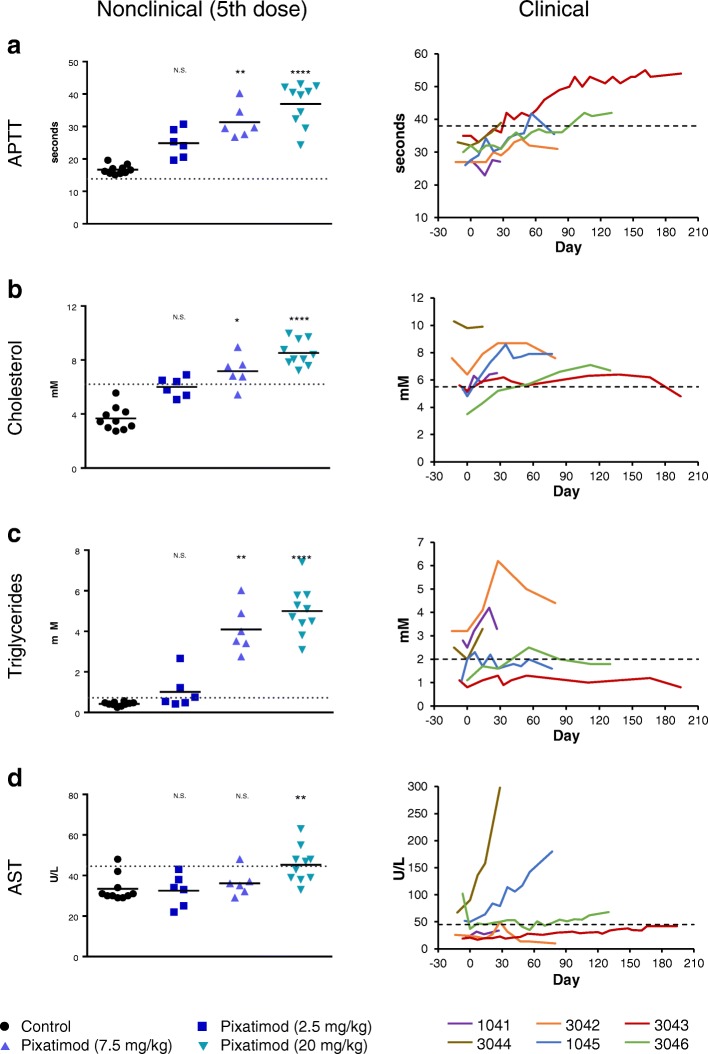
Effect on APTT, blood lipids and AST of weekly IV dosing in beagle dogs and humans (patients in the 100 mg cohort). APTT (**a**), cholesterol (**b**), triglycerides (**c**) and AST (**d**) were measured in the blood of dogs after 5 pixatimod doses (weekly dosing). Treatment averages indicated with short solid horizontal lines. Corresponding data from the six 100 mg patients for these 4 parameters are presented over time. Dotted lines represent upper limit of the normal range for each parameter. * = *P* < 0.05, **P < 0.01, ***P < 0.001, ****P < 0.0001 versus control (Kruskal-Wallis test)

The toxicity profile of pixatimod in beagle dogs compromised of some findings consistent with that of an immunomodulatory agent. To that end, it is of interest to note that significant elevations in body temperature was apparent following the first dose of pixatimod but the effect appeared to dissipate upon repeat dosing by day 30 (Fig. 2a). The other striking effect was the significant increases in large unstained cells (LUCs) following exposure to pixatimod (Fig. 2b). Despite the changes in LUCs, absolute WBC counts and main subsets (lymphocytes, neutrophils and monocytes) remained within normal ranges though changes did, at times, reach statistical significance (Additional file 1). However, significant changes in APTT, cholesterol, triglycerides and AST were noted in the toxicology study (Fig. 3a-d). These toxicology findings were somewhat consistent with reported adverse events in advanced cancer patients (Fig. 3a-d), though elevations in AST were only prominent in two subjects at the maximum tolerated dose (MTD) in some patients but these were unrelated to pixatimod treatment. In the Phase Ia monotherapy clinical trial, some parameters, such as cholesterol and triglycerides, normalised despite repeated exposure to pixatimod suggesting an adaptive response to treatment. Adverse events, severe adverse events and dose limiting toxicities associated with pixatimod treatment in humans have been previously reported [31].

In the toxicology study, there was no mortality or significant changes in bodyweight gain, food consumption, urinalysis, ophthalmologic investigations, ECGs, heart rate, or any local sign at infusion sites during the study (except for oedema in one high dose individual) associated with pixatimod treatment. Pixatimod significantly increased relative weights of liver and kidneys, with modest but dose-dependent decreases in thymus weights but no effect on spleen weight (Additional file 2A-D). Given spleen weights increase following SC dosing in rodents (Additional file 2E), there may be a species difference although the influence of the route of administration cannot be fully discounted as a previous toxicology study in beagles reported non-statistically significant increases in relative spleen weights following treatment with pixatimod (Additional file 2F). Treatment also led to microscopic findings at infusion sites, kidneys, liver, thymus and spleen (Additional file 3). Generally speaking, these dose-dependent alterations were characterized as chronic-active inflammation (the coexistence of chronic inflammation with the presence of mononuclear cell population and superimposed by an acute inflammation with polymorphonuclear cells). Pixatimod also induced dose-dependent hypertrophy of Kupffer cells in the liver and inflammatory changes in the kidneys (multifocal perivascular, mixed cellular infiltrate) with glomerular vacuolation and/or sclerosis noted in high dose groups only. Diffuse mixed cellular infiltrate in the spleen and minimal to mild lymphoid atrophy of the thymus were also observed in mid and high dose animals. No treatment-related changes were reported in the bone marrow smears.

### Comparative exposure (AUC) and route of administration

**Fig. 4:**
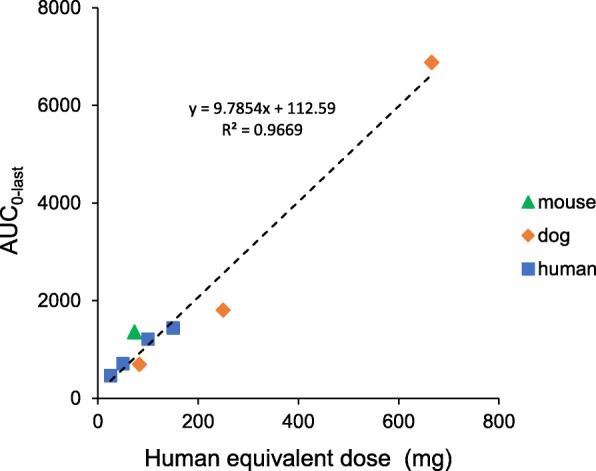
The relationship between a single pixatimod dose and exposure (AUC) across mouse, dog and human. The Week 1 AUC_0-last_ data from Table 1 are plotted as a function of the human equivalent dose (HED). The dotted line represents a linear regression of all of the data yielding an R^2^ of 0.9669 and a y intercept of 112.59

Population-based PK analysis and NCA parameter estimates was reported in the Phase Ia monotherapy study (PG545102) with proportional exposure up to 100 mg dose [31]. Herein, exposure (AUC) following pixatimod in the mouse, dog and in advanced cancer patients is reported in Table 1. Plotting the exposure data from Week 1 from all species (mouse, dog and human) as a function of human equivalent dose (HED) reveals a linear response with a linear regression passing close to the origin (Fig. 4).

**Table 1:** Comparison of mean exposure (AUC0-last) in mouse, dog and human based following intravenous administration of pixatimod

Species	Dose (mg/kg)	HED^a^ / human dose (mg)	AUC_0-last_ (μg.h/mL) ^b^ Week 1	AUC_0-last_ (μg.h/mL) ^b^ Week 4
Mouse	15	73	1358 ^c^	N.D.
Dog	2.5	83	697	1372
	7.5	250	1809	4936
	20	666	6883	10,536
Human	–	25	465	610
	–	50	709	1688
	–	100	1209	2381
	–	150	1441	2781

^a^HED – Human equivalent dose conversion (https://www.fda.gov/downloads/drugs/guidances/ucm078932.pdf)

^b^Area under the pixatimod concentration-time curve from time 0 h (relative to applicable dose) to the last measureable concentration over the dosing interval; derived from the measured concentration values using linear trapezoidal summation

^c^Originally published in [29]

*N.D*. Not Determined

The AUC_0-last_ exposure data for week 4 in patients confirm the accumulation previously reported [31] and is consistent with findings in the dog toxicology study. However, it is unclear whether this is the case in mice as it was impossible to obtain plasma in week 4 (due to the tumor burden in the A2780 xenograft model) and previous data on exposure upon repeated dosing in tumor-bearing immunocompetent mice were not evident across all dose levels, the exposure (AUC) required to achieve efficacy as a monotherapy was 687 μg.h/mL [21] which is in the range of the low dose group (2.5 mg/kg) in the toxicology study and the low dose group (25 mg) in the clinical study by the end of the first cycle (1 month of weekly IV treatment).

**Fig. 5:**
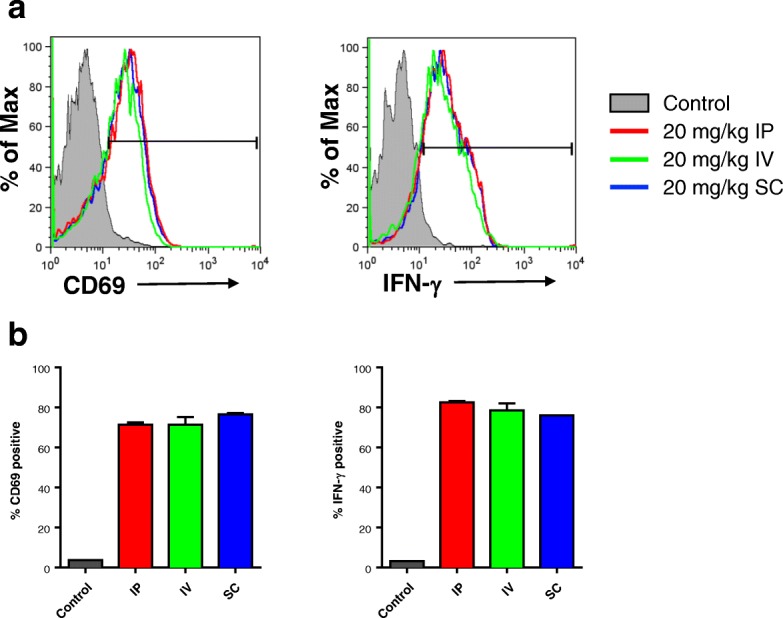
The effect that route of pixatimod administration has on the activation of NK cells. **a** Mice were dosed with 400 μg (20 mg/kg) of pixatimod intraperitoneally (IP), intravenously (IV) or subcutaneously (SC) and after 2 days the activation status of NK cells (CD3^−^, NK1.1^+^) isolated from spleens were assessed. **b** The percentage of NK cells positive for CD69 and IFN-γ are plotted. The gating for CD69 and IFN-γ positive NK cells is shown in panel **a**

In addition to assessing the relationship between dosing and exposure across these species, the efficacy of pixatimod was also examined as a function of the route of administration. After dosing mice via IP, IV and SC routes, pixatimod increased the expression of IFN-γ and CD69 on NK cells to a similar magnitude irrespective of the route, indicating that pixatimod’s immunomodulatory activity is not limited to a particular route of administration (Fig. 5).

### Combination therapy potential

**Fig. 6:**
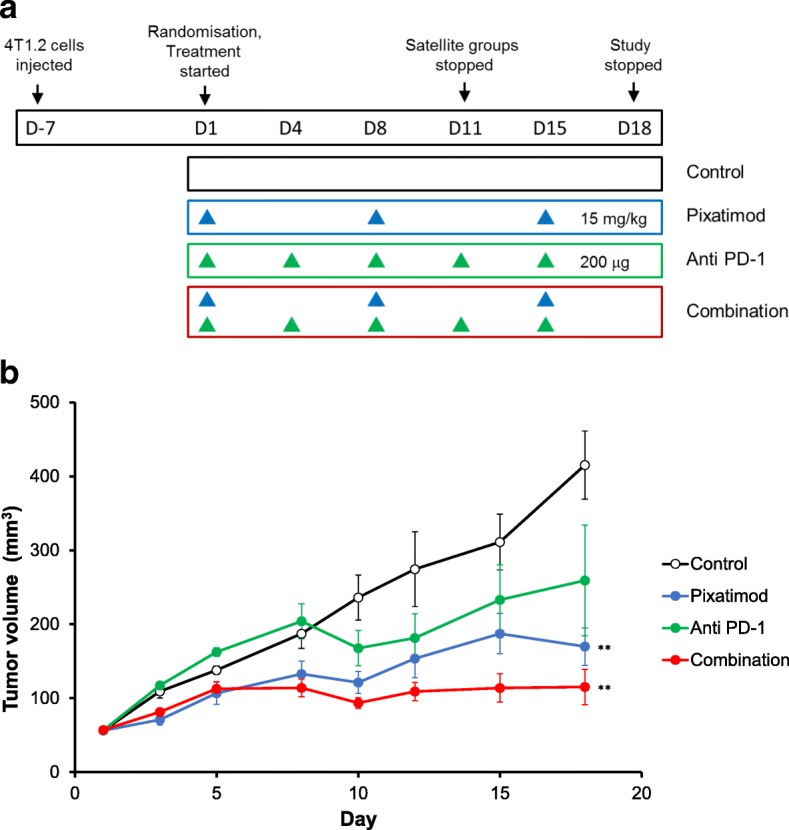
Efficacy of pixatimod in combination with anti-PD-1 in a syngeneic 4T1.2 breast cancer mouse model. **a** Seven days after inoculation, mice were randomised into four treatment groups of six mice each: Control (PBS weekly plus 200 μg isotype antibody twice weekly); Pixatimod (pixatimod 15 mg/kg weekly plus isotype antibody twice weekly); Anti-PD-1 (PBS weekly plus 200 μg anti-PD-1 antibody twice weekly) and Combination (pixatimod weekly plus anti PD-1 antibody twice weekly). Satellite groups of four mice were treated identically and were stopped at day 11 for immune cell analysis (Fig. 6). **b** Tumor volumes were measured throughout the study and the means compared at study conclusion (day 18). **P < 0.01 versus control

Given the multiple modes of immune evasion that cancers have evolved, a significant clinical effort has commenced to evaluate immunotherapies in combination to increase response rates and broaden the types of cancers that can be treated [32]. Even the most successful immunotherapeutic strategy, targeting PD-1, has shown limited utility as a single agent and yields better patient outcomes by utilizing combination strategies [33]. The syngeneic breast cancer model 4T1.2 is considered poorly immunogenic, highly metastatic, and exhibits limited responsiveness to checkpoint blockade [34]. To assess the clinical potential of the pixatimod and anti-PD-1 antibody combination regimen, these agents were tested in the 4T1.2 breast model (Fig. 6a). The combination was significantly more efficacious than the control group or anti-PD-1 treatment alone. Tumor growth in the pixatimod plus isotype antibody (Pixatimod), vehicle plus anti-PD-1 antibody (Anti-PD1) and pixatimod plus anti-PD-1 (Combination) groups was inhibited by 68, 44 and 84%, respectively on day 18. Tumor growth in the pixatimod and combination groups was significantly inhibited on day 18 compared to the vehicle plus isotype antibody (Control) group (Fig. 6b).

**Fig. 7:**
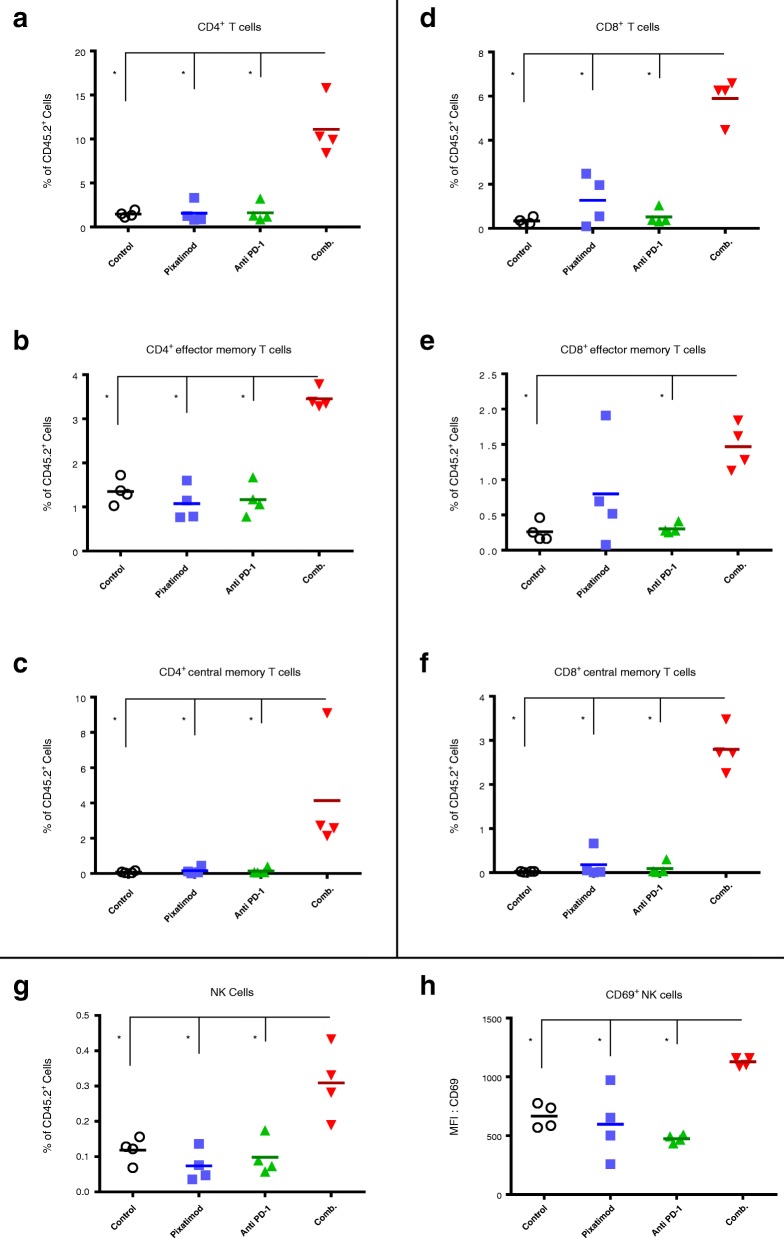
Phenotype analysis of immune cells in the tumors of the 4T1.2 breast cancer model (Fig. 6). Immune cells were isolated from the tumors of mice in the satellite groups (day 11) and phenotyped by flow cytometry. **a** Total CD4^+^ T cells (**b**) effector memory CD4^+^ T cells and (**c**) central memory CD4^+^ T cells. **d** Total CD8^+^ T cells (**e**) effector memory CD8^+^ T cells and (**f**) central memory CD8^+^ T cells. **g** Total and (**h**) CD69^+^ NK cells. Treatment averages indicated with short solid horizontal lines. *P < 0.05 versus control

The tumors of satellite groups from this study were analysed at day 11 of the study for immune cell populations and activation status. The effect of the combination treatment upon intratumoral immunity was striking. The frequency of both CD4^+^ (Fig. 7a) and CD8^+^ (Fig. 7d) T cells were significantly elevated in the tumors of the combination group. Within both the CD4 and CD8 populations, effector memory and central memory cells were increased by the pixatimod-anti-PD-1 combination (Fig. 7b-f). Moreover, both the frequency of bulk NK cells and activated CD69^+^ NK cells were also increased in tumors of the combination group (Fig. 7g and h). In contrast, there was little or no increase in the abundance of CD4^+^ or CD8^+^ T cells or NK cells in the spleens of the mice treated with pixatimod, anti PD-1 antibody or the combination when compared to the spleens of control mice indicating that the immune response was tumor-specific (Additional file 4).

## Discussion

Pixatimod has a unique mechanism of action and a structure that is similarly unconventional in the pharmaceutical world. It targets TAMs via the inhibition of heparanase [9, 10] and it also activates NK cells through TLR9-dependent stimulation of DC [11]. The unique activity is not only attributed to its heparan sulfate (HS) mimetic structure – a recognized approach in the generation of heparanase inhibitors [5–8] – but importantly, to its lipophilic moiety (cholestanol), which sets pixatimod apart from other HS mimetics [1, 3, 21]. The resultant compound is more polar and larger than a typical small molecule drug but has been demonstrated to possess potent antitumor activity in multiple cancer models indicating potential utility in a range of cancers, particularly in combination with other therapeutics. An example of this utility is shown by the efficacy of pixatimod when combined with an anti PD-1 antibody in the 4T1.2 model (Fig. 6), the %TGI for the combination group (84%) was almost two-fold higher compared with the anti-PD-1 antibody group (44%). Though the impact of the combination on 4T1.2 metastasis was not assessed, pixatimod has been previously demonstrated to inhibit spontaneous metastasis and enhance overall survival in this model [22].

In terms of an immune response, synergy was observed in the pixatimod and anti-PD-1 treatment group as significant increases in both tumor-specific CD8 and CD4 effector memory and central memory T cells were evident. Moreover, the combination significantly increased NK cell numbers in the tumors. Though pixatimod alone didn’t significantly increase intratumoral NK cells as previously reported [11], this could be attributed to the different NK-characterizing antibodies (CD49b and CD27 in the 4T1.2 data versus CD335 in the A20 data in [11]) which also may detect different NK cell subsets [35]. Moreover, there may be differences in pixatimod’s ability (as a monotherapy) to increase NK cell infiltration into primary tumors which are disseminated or ‘diffuse’ such as lymphomas compared with transplantable carcinoma models. Finally, pixatimod possesses potent anti-metastatic activity in the 4T1.2 model [22], so it is conceivable that activated NK cells could be directed to distant metastatic sites rather than accumulate in the primary tumor, especially given the critical role of NK cells in the control of spontaneous metastasis in the 4T1.2 model [36].

The key message from this study is that pixatimod, in combination with a PD1 inhibitor, has the potential to enhance a tumor-specific T cell response capable of inhibiting tumor growth, a notion which holds great potential for cancer treatment [37–39]. A number of innate immune activators (including TLR9 agonists) are under development and could prove to be complimentary to T cell based therapies but typically they are administered locally which could limit their utility in the clinic [40, 41]. Thus, pixatimod offers an alternate approach to promote T cell- (and NK cell-) based inflammation in non-inflamed tumors, which is considered one of the biggest challenges in order to expand the subset of patients in whom currently active immunotherapies appear effective [42, 43].

The mechanism whereby the pixatimod/anti-PD-1 combination promotes the recruitment of T cells into tumors is not clear. Pixatimod alone has been shown to activate NK cells which can be detected in spleens and tumors in mouse models but in the same study it showed no activation or recruitment of T cells [11]. However, in combination with anti-PD-1 antibody, pixatimod significantly increases the infiltration of tumor-specific T cells into the TME. This could be related to the blockade of TAMs via pixatimod-mediated heparanase inhibition [9, 10] or maturation of plasmacytoid DCs (pDCs) via TLR9 [44] resulting in the diminution of the tolerogenic signalling environment associated with TAM, other myeloid cells or immature pDCs, particularly in cold tumors. Though the first mechanism could lead to synergy with anti-PD1 agents due to reduced numbers of immunosuppressive PD-L1/2^+^ cells in the TME, such as tolerogenic myeloid cells or tumor cells, the second mechanism via TLR9 [11] is equally, if not more likely to work in concert with PD-1 blockade. TLR9 agonists are known to increase the efficacy of anti-PD-1 agents in preclinical models [45, 46] and can involve the polarization of naive macrophages toward a M1-like phenotype [47]. Conversely, it is known that depletion of TAMs or M2 macrophages using CSF-1R inhibition enhances DC immunotherapy [48] and checkpoint inhibition [49]. Viewing M1 macrophages and DC as essentially antigen presenting cells [19], we conclude that at least in combination with a PD-1 inhibitor, pixatimod’s immunomodulatory activity (whether this is a direct stimulation of DC or a polarization from M2 to M1 macrophages) leads to improved immune recognition of tumor cells as shown by the synergistic increases in T cell infiltration into the TME.

Given the potent immune stimulatory activity of pixatimod, it is important to characterize toxicologic responses that could be associated with excessive activation of the immune system. Upon initial exposure to pixatimod in beagle dogs, the elevations in body temperature and LUCs are particularly noteworthy. Flu-like symptoms (including fever) have been previously reported as a response to innate immune activators such as TLR9 agonists [44] but in this instance at least, elevated temperatures dissipated upon repeated dosing suggesting an adaptive response. Similarly, pixatimod-induced elevations in percent LUCs – defined as atypical large lymphocytes or monocytic cells that may increase with an inflammatory response [50] – declined, at least in the high dose group, upon repeated exposure. In the PG545102 monotherapy trial, pixatimod induces flu-like symptoms in patients at doses at or above 50 mg, which required prophylactic paracetamol [31], but LUCs could not be measured at hospital sites.

The major nonclinical toxicities associated with pixatimod were elevated cholesterol and triglyceride levels, increases in relative weights of liver and kidney, cellular infiltrates in liver, kidney, and spleen, hypertrophy of Kupffer cells, tubular dilatation and glomerular vacuolation and/or sclerosis. Though relative spleen weights significantly increase following pixatimod treatment in rodents following SC dosing, this finding was not significant in the SC dog toxicology study and not apparent whatsoever in the IV dog study. So, while TLR9 expression may be lower in dog or human macrophages than mouse or rat macrophages [51], the route of administration could also contribute to this effect. This is potentially relevant for two reasons. First, given that pixatimod’s immunostimulatory may be mediated via TLR9 [11], but note that pixatimod is not a CpG oligonucleotide (ODN) or a TLR9 agonist, this may also account for the fact that the histopathological findings for CpG-ODN [51] were not reported in the pixatimod toxicology studies. Second, the clinical route of administration is now via the IV route and though the safety profile appears promising, the immunostimulatory effect on NK cells is equivalent to other route of administration. In addition to the hyperlipidemia and vacuolation, other changes such as decreased RBC, HGB, HCT, lymphocytes and platelets and increases in percent neutrophils and APTT were considered toxicologically relevant. In contrast, these parameters were not clinically significant in patients though hyperlipidema and elevated APTT were considered to be related to pixatimod treatment. Taken together, the safety profile of pixatimod is consistent with an innate immune activator which has the potential to induce an inflammatory response in the host.

The PK profile of pixatimod has been previously described in mouse [29] and human subjects [31] but herein the relationship between pixatimod dose and exposure (after the first dose) was found to be linear across mouse, dog and human (Fig. 4). This indicates, firstly, that exposure, at least for the initial pixatimod dose, is predictably proportional to the dose administered and, secondly, that the assumptions inherent in extrapolation from animal efficacy and toxicology studies to humans appear valid and may be utilised for continuing PK/PD analyses during development.

In the Phase Ia monotherapy trial, 20 patients experienced treatment emergent adverse events (AE) that were possibly, likely, or certainly related to pixatimod. The majority of AEs in this category were associated with infusion reactions: chills, pyrexia, infusion related reactions, and hypertension. In terms of clinical activity, 16 patients had efficacy assessments during pixatimod treatment and six of these had stable disease (SD) at 8 weeks as measured by RECIST 1.1 criteria. This ratio, six of sixteen assessed, represents a 38% disease control rate at 8 weeks. The estimated median duration of SD for patients on the study was 57 days [31]. Based on the immunomodulatory properties of pixatimod and emerging preclinical data in combination with an anti-PD-1 antibody, a new clinical trial is underway in Australia investigating pixatimod in combination with nivolumab (Opdivo®) in patients with advanced solid tumors with an expansion cohort in patients with pancreatic adenocarcinoma.

## Conclusions

Pixatimod modulates macrophages and DCs to activate NK cells but in this study it has also been demonstrated to enhance the antitumor activity of a PD-1 inhibitor, an effect that correlates with increased frequency of T cells and NK cells within the TME. The safety profile indicated that it has mild inflammatory properties but the compound was well tolerated up to 100 mg in the monotherapy clinical trial. Pixatimod is currently under investigation in a Phase Ib study for advanced cancer/pancreatic adenocarcinoma with the checkpoint inhibitor nivolumab (Opdivo®).

## Additional files

Additional file 1: Effect on total white blood cells (WBC), lymphocytes, neutrophils and platelets of weekly IV dosing in beagle dogs and humans (patients in the 100 mg cohort). WBC (A), lymphocytes (B), neutrophils (C), monocytes (D) and platelets (E) were measured in the blood of dogs after 5 pixatimod doses (weekly dosing). Treatment averages indicated with short solid horizontal lines. Corresponding data from the six 100 mg patients for these 4 parameters are presented over time. Dotted lines represent limits of the normal range for each parameter. *P < 0.05, **P < 0.01, ***P < 0.001, ****P < 0.0001 versus control (Kruskal-Wallis test). (PPTX 142 kb)

Additional file 2: Effect of pixatimod dosing on selected organ weights in beagle dogs (IV and SC) and rats (SC). Repeated weekly IV exposure (× 5) of pixatimod increases the relative weights of liver (A) and kidney (B) but not thymus (C) nor spleen (D) in beagle dogs. Relative weights of liver and kidneys were significantly increased in the high dose group (20 mg/kg) with the mid-dose group (7.5 mg/kg) also significantly increasing the relative weight of kidneys. Data shown is for combined sexes. Relative spleen weight was increased in a dose-dependent manner after repeated weekly SC exposure (× 5) in rats (E). Study size, *n* = 15 for control and 48 mg/kg groups and *n* = 10 for 3 and 12 mg/kg groups. As seen with IV exposure (D), repeated weekly SC exposure (× 5) in beagle dogs had no significant effect on spleen weight (F). Study size, *n* = 5 for control and 20 mg/kg groups and *n* = 3 for 2.5 and 7.5 mg/kg groups. *P < 0.05, **P < 0.01, ***P < 0.001 versus control (Kruskal-Wallis test for the dog study, ANOVA followed by Dunnett’s comparison for the rat study). (PPTX 65 kb)

Additional file 3: The incidence and severity of noteworthy microscopic findings in kidneys, liver, spleen and thymus. Perivascular mixed cell infiltrate was present in most mid- and high-dose individuals. Minimal to mild dilatation of renal tubules was apparent in most treated individuals though minimal or mild glomerular vacuolation or sclerosis was only reported in high-dose individuals. Minimal to mild hepatocellular hypertrophy was evident across dose levels whereas incidence and severity of hypertrophy of Kupffer cells in the liver was dose-dependent. A minimal to mild increase in cell infiltrate was apparent some high-dose individuals. There was also evidence of diffuse mixed cellular infiltrate in the spleen and minimal to mild lymphoid atrophy of the thymus observed in mid and high dose animals. (DOCX 13 kb)

Additional file 4: Phenotypic analysis of CD4^+^ and CD8^+^ T cells, and NK cells, in the spleens of the 4T1.2 breast cancer model. Splenocytes were isolated in the satellite groups (day 11) and assessed by flow cytometry. (A) Total CD4^+^ T cells (B) Total CD8^+^ T cells (C) Total NK cells. Spleens of all four mice from each treatment group were combined for analysis. (PPTX 47 kb)

## Abbreviations

AE: Adverse event
APTT: Activated partial thromboplastin time
AST: Aspartate transaminase
AUC: Area under the curve
DC: Dendritic cells
FDA: Food and Drug Administration
HCT: Hematocrit
HED: Human equivalent dose
HGB: Hemoglobin
IND: Investigational new drug
IP: Intraperitoneal
IV: Intravenous
LUC: Large unstained cells
MTD: Maximum tolerated dose
NK (cells): Natural killer cells
PK: Pharmacokinetics
RBC: Red blood cells
RECIST: Response evaluation criteria in solid tumors
SC: Subcutaneous
SD: Stable disease
TAM: Tumor-associated macrophages
TME: Tumor micro-environment
WBC: White blood cells
