# Supplementary material for: Immunomodulatory activities of pixatimod: emerging nonclinical and clinical data, and its potential utility in combination with PD-1 inhibitors
Source: J Immunother Cancer. 2018 Jun 14;6:54. doi: 10.1186/s40425-018-0363-5 (PMC6000956; doi:10.1186/s40425-018-0363-5)
Supplement: Supplementary file 1 — Effect on total white blood cells (WBC), lymphocytes, neutrophils and platelets of weekly IV dosing in beagle dogs and humans (patients in the 100 mg cohort). WBC (A), lymphocytes (B), neutrophils (C), monocytes (D) and platelets (E) were measured in the blood of dogs after 5 pixatimod doses (weekly dosing). Treatment averages indicated with short solid horizontal lines. Corresponding data from the six 100 mg patients for these 4 parameters are presented over time. Dotted lines represent limits of the normal range for each parameter. *P < 0.05, **P < 0.01, ***P < 0.001, ****P < 0.0001 versus control (Kruskal-Wallis test). (PPTX 142 kb) [file 40425_2018_363_MOESM1_ESM.pptx]

## Slide 1
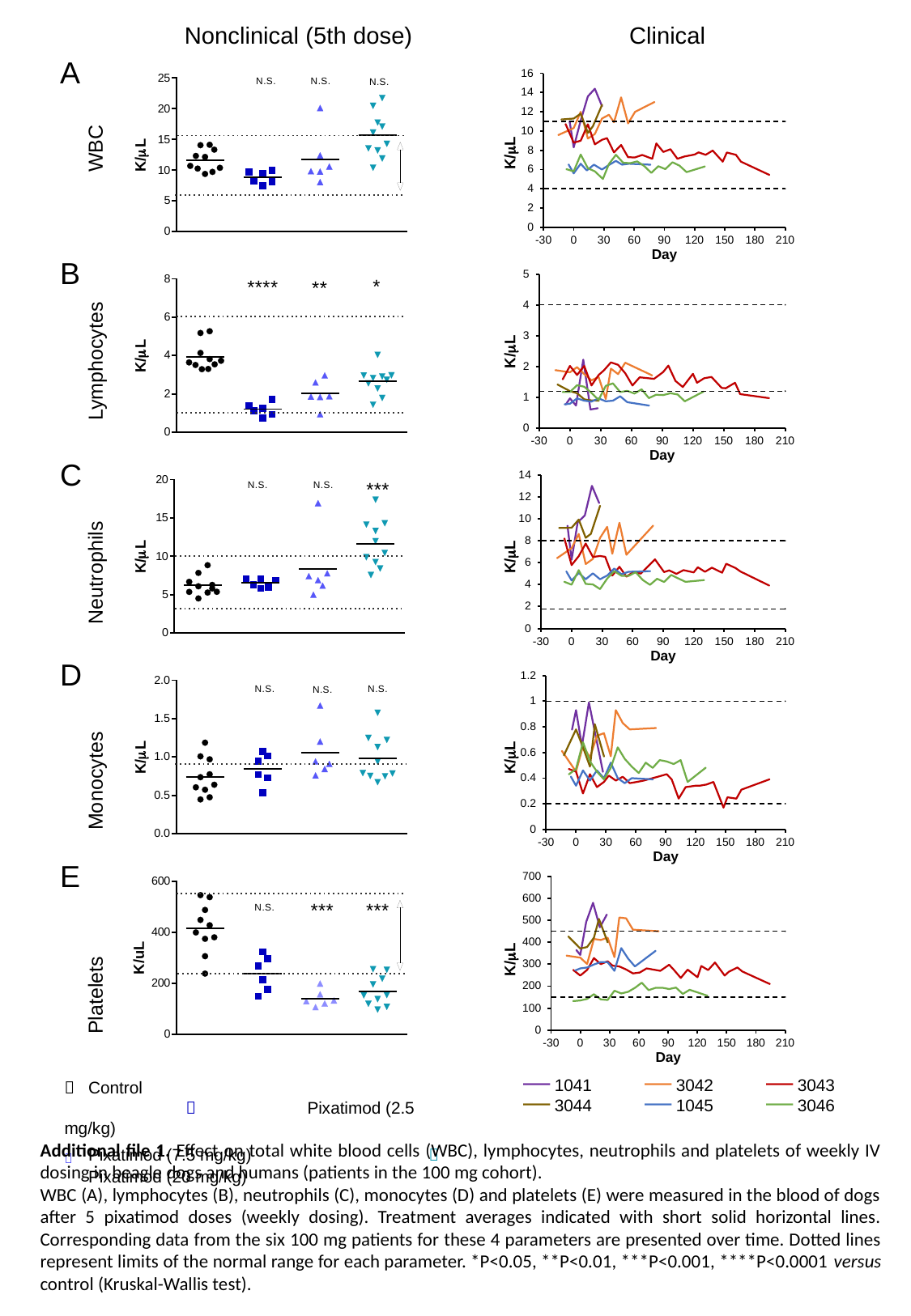

Nonclinical (5th dose)
Clinical
A
WBC
B
Lymphocytes
C
Neutrophils
D
Monocytes
E
Platelets
	Control							Pixatimod (2.5 mg/kg)
	Pixatimod (7.5 mg/kg)			Pixatimod (20 mg/kg)
— 1041 	— 3042 	— 3043
— 3044 	— 1045 	— 3046
Additional file 1. Effect on total white blood cells (WBC), lymphocytes, neutrophils and platelets of weekly IV dosing in beagle dogs and humans (patients in the 100 mg cohort).
WBC (A), lymphocytes (B), neutrophils (C), monocytes (D) and platelets (E) were measured in the blood of dogs after 5 pixatimod doses (weekly dosing). Treatment averages indicated with short solid horizontal lines. Corresponding data from the six 100 mg patients for these 4 parameters are presented over time. Dotted lines represent limits of the normal range for each parameter. *P<0.05, **P<0.01, ***P<0.001, ****P<0.0001 versus control (Kruskal-Wallis test).
