# Supplementary material for: Immunomodulatory activities of pixatimod: emerging nonclinical and clinical data, and its potential utility in combination with PD-1 inhibitors
Source: J Immunother Cancer. 2018 Jun 14;6:54. doi: 10.1186/s40425-018-0363-5 (PMC6000956; doi:10.1186/s40425-018-0363-5)
Supplement: Supplementary file 2 — Effect of pixatimod dosing on selected organ weights in beagle dogs (IV and SC) and rats (SC). Repeated weekly IV exposure (× 5) of pixatimod increases the relative weights of liver (A) and kidney (B) but not thymus (C) nor spleen (D) in beagle dogs. Relative weights of liver and kidneys were significantly increased in the high dose group (20 mg/kg) with the mid-dose group (7.5 mg/kg) also significantly increasing the relative weight of kidneys. Data shown is for combined sexes. Relative spleen weight was increased in a dose-dependent manner after repeated weekly SC exposure (× 5) in rats (E). Study size, n = 15 for control and 48 mg/kg groups and n = 10 for 3 and 12 mg/kg groups. As seen with IV exposure (D), repeated weekly SC exposure (× 5) in beagle dogs had no significant effect on spleen weight (F). Study size, n = 5 for control and 20 mg/kg groups and n = 3 for 2.5 and 7.5 mg/kg groups. *P < 0.05, **P < 0.01, ***P < 0.001 versus control (Kruskal-Wallis test for the dog study, ANOVA followed by Dunnett’s comparison for the rat study). (PPTX 65 kb) [file 40425_2018_363_MOESM2_ESM.pptx]

## Slide 1
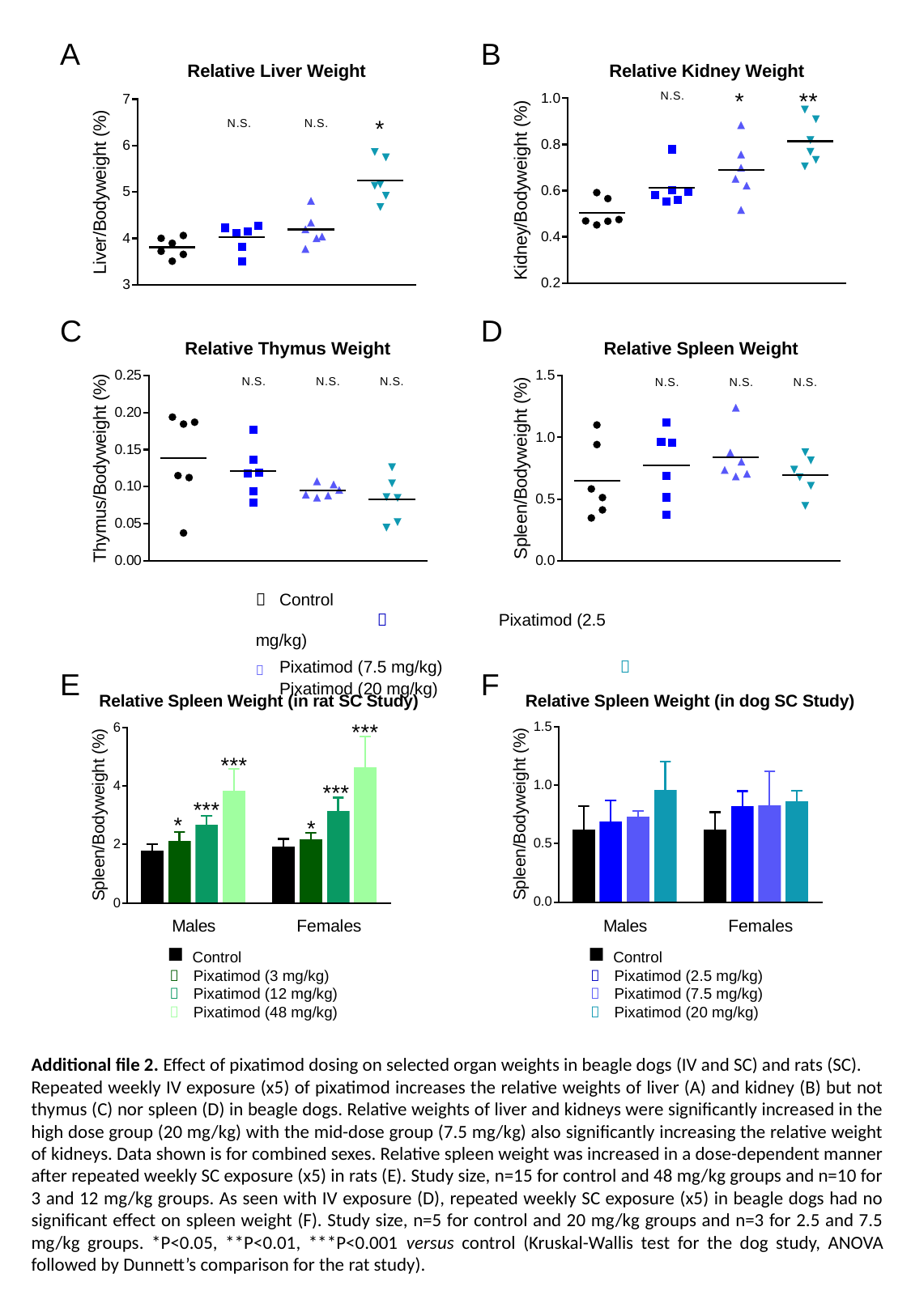

A
B
C
D
	Control							Pixatimod (2.5 mg/kg)
	Pixatimod (7.5 mg/kg)			Pixatimod (20 mg/kg)
E
F
Control
	Pixatimod (3 mg/kg)
	Pixatimod (12 mg/kg)
	Pixatimod (48 mg/kg)
Control
	Pixatimod (2.5 mg/kg)
	Pixatimod (7.5 mg/kg)
	Pixatimod (20 mg/kg)
Additional file 2. Effect of pixatimod dosing on selected organ weights in beagle dogs (IV and SC) and rats (SC).
Repeated weekly IV exposure (x5) of pixatimod increases the relative weights of liver (A) and kidney (B) but not thymus (C) nor spleen (D) in beagle dogs. Relative weights of liver and kidneys were significantly increased in the high dose group (20 mg/kg) with the mid-dose group (7.5 mg/kg) also significantly increasing the relative weight of kidneys. Data shown is for combined sexes. Relative spleen weight was increased in a dose-dependent manner after repeated weekly SC exposure (x5) in rats (E). Study size, n=15 for control and 48 mg/kg groups and n=10 for 3 and 12 mg/kg groups. As seen with IV exposure (D), repeated weekly SC exposure (x5) in beagle dogs had no significant effect on spleen weight (F). Study size, n=5 for control and 20 mg/kg groups and n=3 for 2.5 and 7.5 mg/kg groups. *P<0.05, **P<0.01, ***P<0.001 versus control (Kruskal-Wallis test for the dog study, ANOVA followed by Dunnett’s comparison for the rat study).
