# Supplementary material for: Immunomodulatory activities of pixatimod: emerging nonclinical and clinical data, and its potential utility in combination with PD-1 inhibitors
Source: J Immunother Cancer. 2018 Jun 14;6:54. doi: 10.1186/s40425-018-0363-5 (PMC6000956; doi:10.1186/s40425-018-0363-5)
Supplement: Supplementary file 3 — The incidence and severity of noteworthy microscopic findings in kidneys, liver, spleen and thymus. Perivascular mixed cell infiltrate was present in most mid- and high-dose individuals. Minimal to mild dilatation of renal tubules was apparent in most treated individuals though minimal or mild glomerular vacuolation or sclerosis was only reported in high-dose individuals. Minimal to mild hepatocellular hypertrophy was evident across dose levels whereas incidence and severity of hypertrophy of Kupffer cells in the liver was dose-dependent. A minimal to mild increase in cell infiltrate was apparent some high-dose individuals. There was also evidence of diffuse mixed cellular infiltrate in the spleen and minimal to mild lymphoid atrophy of the thymus observed in mid and high dose animals. (DOCX 13 kb) [file 40425_2018_363_MOESM3_ESM.docx]

**Additional File 3:** The incidence and severity of noteworthy microscopic findings in kidneys, liver, spleen and thymus.

Perivascular mixed cell infiltrate was present in most mid- and high-dose individuals. Minimal to mild dilatation of renal tubules was apparent in most treated individuals though minimal or mild glomerular vacuolation or sclerosis was only reported in high-dose individuals. Minimal to mild hepatocellular hypertrophy was evident across dose levels whereas incidence and severity of hypertrophy of Kupffer cells in the liver was dose-dependent. A minimal to mild increase in cell infiltrate was apparent some high-dose individuals. There was also evidence of diffuse mixed cellular infiltrate in the spleen and minimal to mild lymphoid atrophy of the thymus observed in mid and high dose animals.

|  |  | Group | | | |
| --- | --- | --- | --- | --- | --- |
|  |  | Control | Low Dose | Mid Dose | High Dose |
| No. of animals examined | | 6 | 6 | 6 | 6 |
| **Kidney Dilatation tubular**  minimal  mild  **Vacuolation glomerular**  mild  **Sclerosis glomerular**  minimal  mild  **Cell infiltrate, mixed, perivascular**  minimal  mild  moderate | | **0**  **0**  **0**  **0** | **4**  4  **0**  **0**  **1**  1 | **6**  5  1  **0**  **0**  **5**  4  1 | **6**  6  **4**  4  **3**  2  1  **6**  1  4  1 |
| **Liver Hypertrophy hepatocellular**  minimal  mild  **Hypertrophy, Kupffer cells**  minimal  mild  **Cell infiltrate, mixed, diffuse**  mild  **Cell infiltrate, mononuclear, multifocal**  minimal | | **0**  **0**  **0**  **1**  1 | **5**  2  3  **2**  1  1  **0**  **0** | **6**  1  5  **6**  4  2  **0**  **0** | **6**  1  5  **6**  2  4  **2**  2  0 |
| **Spleen Cell infiltrate, mixed cellular, diffuse minimal** | | **0** | **0** | **2** | **4** |
| **Thymus Lymphoid atrophy**  minimal  mild | | **0** | **0** | **0** | **2**  1  1 |
