# Supplementary material for: Immunomodulatory activities of pixatimod: emerging nonclinical and clinical data, and its potential utility in combination with PD-1 inhibitors
Source: J Immunother Cancer. 2018 Jun 14;6:54. doi: 10.1186/s40425-018-0363-5 (PMC6000956; doi:10.1186/s40425-018-0363-5)
Supplement: Supplementary file 4 — Phenotypic analysis of CD4+ and CD8+ T cells, and NK cells, in the spleens of the 4T1.2 breast cancer model. Splenocytes were isolated in the satellite groups (day 11) and assessed by flow cytometry. (A) Total CD4+ T cells (B) Total CD8+ T cells (C) Total NK cells. Spleens of all four mice from each treatment group were combined for analysis. (PPTX 47 kb) [file 40425_2018_363_MOESM4_ESM.pptx]

## Slide 1
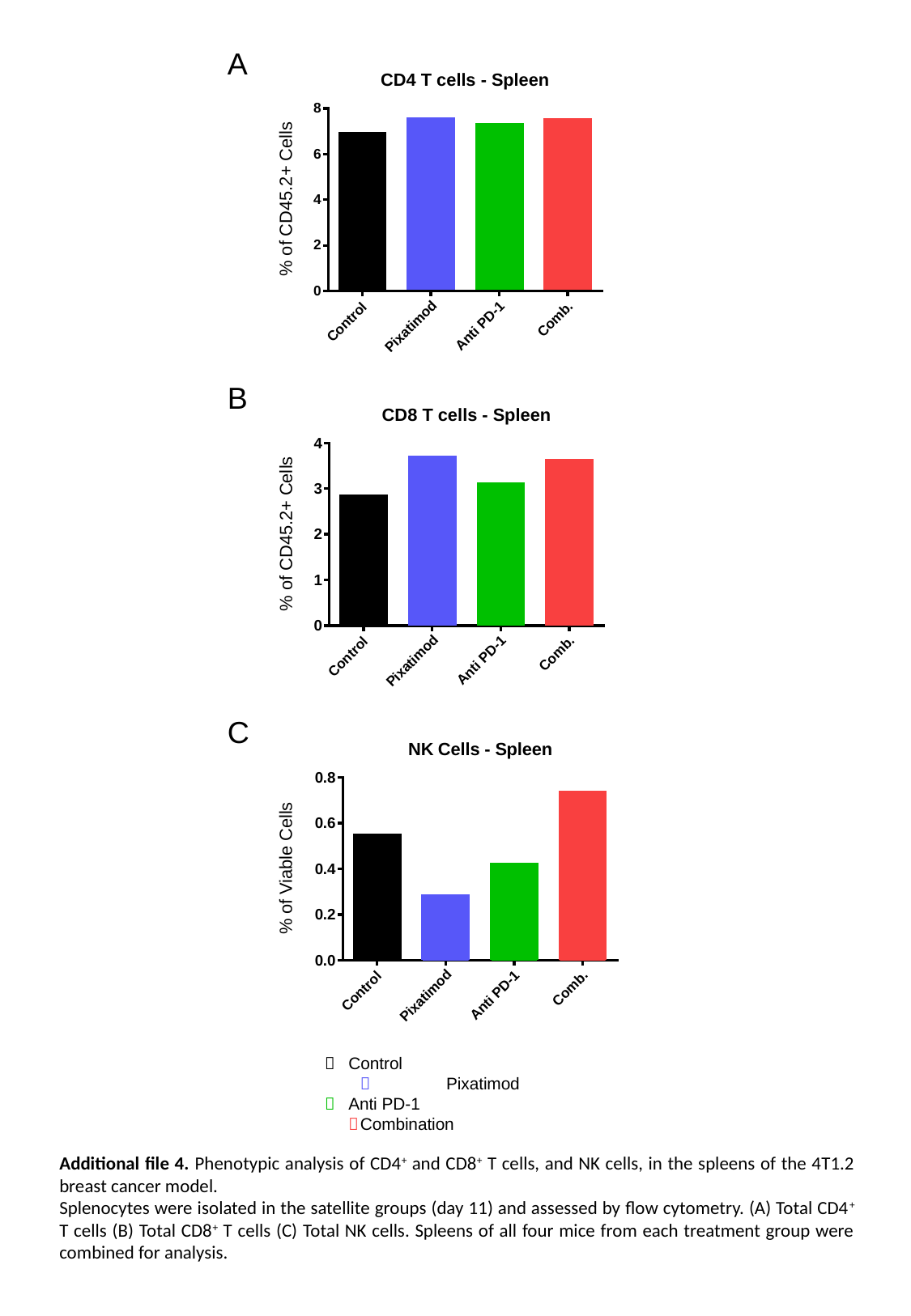

A
B
C
	Control					Pixatimod
	Anti PD-1				Combination
Additional file 4. Phenotypic analysis of CD4+ and CD8+ T cells, and NK cells, in the spleens of the 4T1.2 breast cancer model.
Splenocytes were isolated in the satellite groups (day 11) and assessed by flow cytometry. (A) Total CD4+ T cells (B) Total CD8+ T cells (C) Total NK cells. Spleens of all four mice from each treatment group were combined for analysis.
